# A Comparative Analysis of Tooth Size Discrepancy between Male and Female Subjects Presenting with a Class I Malocclusion

**DOI:** 10.1155/2018/7641908

**Published:** 2018-07-15

**Authors:** Eva Man Yee Leung, Yanqi Yang, Balvinder Khambay, Ricky Wing Kit Wong, Colman McGrath, Min Gu

**Affiliations:** ^1^Orthodontics, Faculty of Dentistry, The University of Hong Kong, 34 Hospital Road, Hong Kong; ^2^College of Medical and Dental Sciences, The School of Dentistry, University of Birmingham, Birmingham, UK; ^3^Department of Dentistry and Maxillofacial Surgery Cleft Center (Craniofacial Orthodontics), United Christian Hospital, 130 Hip Wo Street, Kwun Tong, Kowloon, Hong Kong; ^4^Dental Public Health, Faculty of Dentistry, The University of Hong Kong, 34 Hospital Road, Hong Kong

## Abstract

**Objectives:**

To evaluate the tooth size discrepancy and Bolton's ratios between male and female subjects with a Class I malocclusion.

**Materials and Methods:**

The digital e-models of 100 male and 100 female 12-year-old southern Chinese children with a Class I malocclusion were selected. The mesiodistal widths from permanent first molar to the contralateral side first molar of the upper and lower dentitions were measured. Differences between the tooth size discrepancy, together with the anterior and overall Bolton's ratios between male and female subjects, were assessed using a two-sample* t*-test. A paired* t*-test was used to determine differences between antimetric pairs of teeth within the same arch.

**Results:**

Females had statistically significant smaller teeth than males (*P *< 0.05) except the upper left and lower left lateral incisor and lower left and right central incisors. The mean values of anterior Bolton's ratios for males and females were 77.04 and 77.03, respectively (*P *> 0.05), while the mean values of overall Bolton's ratios of male and female are 90.48 and 90.65, respectively (*P *< 0.05). The clinical significant differences (Cohen's d > 0.2) for contralateral tooth size were shown on the maxillary canines, lateral incisors, and central incisors of males; and mandibular canines and lateral incisors of females.

**Conclusions:**

Southern Chinese females presenting with Class I malocclusions have smaller mesiodistal tooth dimensions compared to males. Both males and females presented several tooth size asymmetries. There are no statistical differences in anterior and overall Bolton's ratios between the genders.

## 1. Introduction

The maxillary to mandibular tooth size relationship is important to achieve ideal overjet, overbite, and occlusal interdigitation following orthodontic treatment and is often referred to as the “seventh key” to an ideal occlusion [1]. A tooth size discrepancy can affect the final outcome and stability of orthodontic treatment [2, 3]. Previous studies have shown a correlation between the mesiodistal tooth widths of maxillary and mandibular teeth in Caucasians [4–6]. Ratios for the estimation of tooth size discrepancy have been reported as the “Bolton's standards” [2]. The first ratio, the anterior ratio, is obtained by measuring the summed mesiodistal widths of the mandibular to maxillary anterior teeth, while the second or overall ratio is the summation of mesiodistal widths of all mandibular to maxillary teeth from first molar to first molar.

Previous studies have compared tooth size between males and females of individuals with Class I malocclusions from America, Egypt, and Mexico [7]. The total and anterior Bolton ratios have been reported greater in males than in females, in a British population and in a black, Hispanic, and white population [8, 9], while others have reported no differences in anterior or posterior tooth size proportions between males and females in black North American and a Saudi population [10, 11].

Besides ethnicity influencing tooth size, studies have reported that tooth size discrepancies may vary among different malocclusion groups. A study based on southern Chinese children validated the use of Bolton standards in individuals with a Class I occlusion but not those with Class II or Class III malocclusions [12]. A recent study confirmed a highest percentage of clinically significant tooth size discrepancy in Class II and Class III surgical cases [13]. Interestingly another Chinese population study showed there was a higher frequency of tooth size discrepancy in Class III malocclusions compared to other malocclusion types [14].

Differences in dental morphology in the Southern Chinese and Hong Kong population have previously been reported [15]. There was a higher prevalence of hyperdontia (2.6%), congenitally missing mandibular incisors (5.6%), dens evaginatus (4.7%), and double tooth (0.8%) than reported in Caucasians. In addition, there was a higher prevalence of semi-shovel shaped maxillary incisors (45.6%) and protostylid cusps on mandibular molars (37.5%) in the Southern Chinese and Hong Kong population. Some of these localized dental morphological variations may have an effect on the Bolton ratio. Therefore Bolton's ratios may not be applicable for both genders, across populations or between malocclusions.

This research aimed to compare the tooth size, as well as the Bolton anterior and overall ratios between male and female southern Chinese individuals presenting with a Class I malocclusion. The null hypothesis is that there is no difference in tooth size and Bolton ratio between male and female southern Chinese individuals presenting with a Class I malocclusion.

## 2. Materials and Methods

### 2.1. Sampling

A sample of orthodontic digital e-models of 609 random 12-year-old southern Chinese children was obtained as a part of data in a cross-sectional oral health survey named “Children of 1997” conducted at the Faculty of Dentistry, the University of Hong Kong. This sample was recruited from 45 secondary schools (accounting for about 10% of all local secondary schools in Hong Kong) from 18 districts of Hong Kong. From the 609 digital models, 100 males and 100 females were selected as having a Class I malocclusion based on the British Standard Institution incisor relationship and met the following inclusion criteria [16]:

- Permanent dentition present (excluding third molars)
- No previous or current active orthodontic treatment
- No impacted teeth
- No hypodontia
- No carious teeth, teeth with interproximal restoration, or fractured teeth
- No abnormal tooth morphology

This study was approved by the Institutional Review Board of the University of Hong Kong/Hospital Authority Hong Kong West Cluster (UW 09-453).

### 2.2. Sample Size Calculation

The estimation of sample size was calculated by running the G*∗*Power 3.17 (Franz Faul, University Kiel, Germany 2013) software. A sample size of 200 subjects is required in order to detect clinically significant difference based on an Altman nomogram with a power of 0.8 at* P *< 0.05; standard deviation and standardized difference are 1.0 and 0.4, respectively [17].

### 2.3. Digital Models Measurement

**Figure 1:**
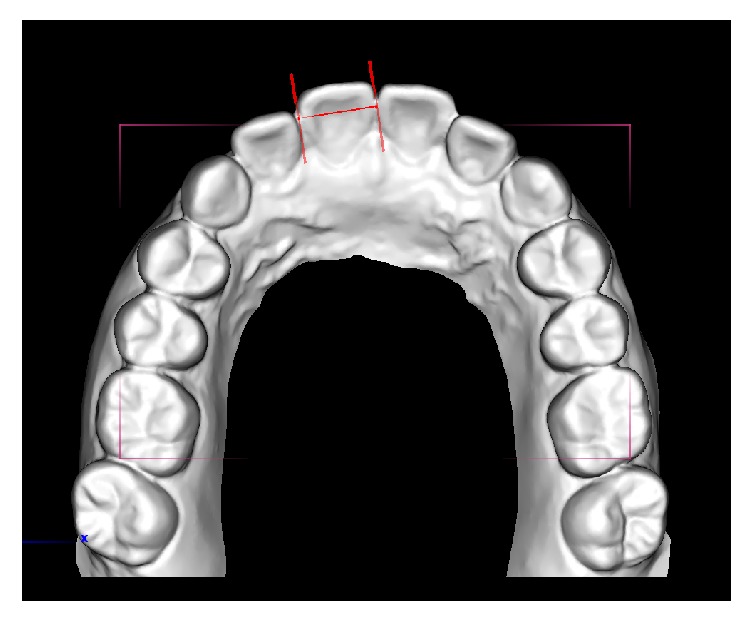
Measurement of tooth width using O3DM digital model software.

Each patient's e-model was imported and viewed in O3DM digital model software programme (Version 3.2.1, Ortholab 2003-2012, Poland). Definitions of mesiodistal crown width based on criteria of Seipel [18]. The e-models were enlarged and rotated into different angulation to allow more precise identification of the mesial and distal contact points along the occlusal surfaces of each tooth [19]. After calibration with another orthodontist (YY) experienced in e-model measurement, the same operator (EL) measured the mesiodistal width of the teeth from incisors, canines, premolars, and first molars in both maxillary and mandibular arches using the appropriate measurement tool incorporated in the software (Figure 1). Individual tooth mesiodistal tooth width and anterior and overall Bolton's ratios were calculated.

Anterior and overall Bolton's ratios were calculated with the following formula:

(1) Anterior  Bolton's  ratio=Sum  of  mandibular  3-3Sum  of  maxillary  3-3×100Overall  Bolton's  ratio=Sum  of  mandibular  6-6Sum  of  maxillary  6-6×100

### 2.4. Intraoperator Error

Twenty sets of e-models chosen at random were selected for intraoperator error assessment. The mesiodistal widths of each tooth from first molar to contralateral first molar for upper and lower arches were measured twice with a 2-week interval by the same observer (EL). The intraclass correlation coefficient (ICC) was 0.90, indicating that the reliability of these measurements was satisfactory.

In our another study [20], digital model measurements have been compared to plaster cast measurements, and the effect sizes (Cohen's d) were generally less than 0.2, which showed the validity of digital model measurement was also satisfactory.

### 2.5. Statistics

Following assessment of normal distribution using the Shapiro-Wilk test, a two-sample* t-*test was used to compare the mean of each tooth width and the mean of anterior and overall Bolton's ratio between male and female subjects; a parted* t*-test was used to test the symmetry of tooth size between contralateral teeth. All the statistical analyses were performed using the Statistical Package for Social Science software (IBM SPSS Statistics 20, IBM Corp., USA).

## 3. Results

### 3.1. Comparison of Tooth Size

Table 1 shows that females had statistically significant (*P* < 0.05) smaller teeth than males except the following teeth: upper left and lower left lateral incisor and lower left and right central incisors. However, the mean differences of all female and male teeth size were less than or equal to 0.2mm except the canines and first molars. The 95% confidence intervals for the mean differences for males and females were greater than 0.5 mm for all four first molars and 0.4 mm for all of canines.

### 3.2. Comparison of Bolton's Ratio

The mean values for the anterior Bolton's ratio of males and females were 77.04 ± 1.86 and 77.03 ± 1.86, respectively; this was not statistical significant (*P* > 0.05). When considering the overall Bolton's ratios, the mean values of male and female are 90.48 ± 1.79 and 90.65 ± 1.60, respectively; again this was not statistically significant (*P* > 0.05).

### 3.3. Comparison of Contralateral Tooth Size of the Same Gender

Table 2 shows that the maxillary canines, lateral incisors, and central incisors of males as well as the mandibular canines and lateral incisors in the female group showed a Cohen's d value between 0.2 and 0.5. Values below 0.2 indicate that mean differences in mesiodistal width between the antimetric pair may not be clinically significant [21].

## 4. Discussion

Although diagnostic measurements have traditionally been based on plaster dental casts, with the advancement of 3D digital imaging technology, e-models are a valid alternative [22]. The accuracy of space analysis evaluation, tooth width measurements, and arch relationships on digital e-models is clinically acceptable and reproducible when compared with traditional plaster study model analysis [23, 24]. Moreover, digital measurement is more rapid and less variable than the manual method with needle-point dividers or Boley gauge (Vernier caliper) [25, 26].

Given that tooth size discrepancies may be influenced by malocclusion type and ethnicity, the sample for this study was selected from a homogenous population of Hong Kong individuals with Class I incisor relationships. We found that females had statistically significant (*P *< 0.05) smaller teeth than males with the exception of the upper left and right lateral incisors, lower left lateral incisor, and lower left and right central incisors. This was in agreement with a previous study which reported that southern Chinese males tooth dimension in general was 2.9% wider in comparison to females in a random sample irrespective of malocclusion [27]. However, the mean differences between female and male tooth size was less than 0.15mm, implying this was probably not clinically significant. This finding was comparable to Townsend who reported that the mesiodistal dimensions of maxillary first and second premolars showed sexual dimorphism, with the mean male premolar being wider than female [28]. Morrees [19] also reported that permanent canines were on average 6% larger in males for both mesiodistal and buccolingual dimensions. Interestingly, the distribution of the teeth involved in the various studies was different. One could surmise that generally if males have larger teeth than females, and there is no difference in lower incisors widths, then males could have smaller incisors than expected perhaps linking this to the higher prevalence of congenitally missing mandibular incisors (5.6%) in the Hong Kong population [29]. The aetiology of these findings requires further investigation.

One of the proposed aetiologies of tooth anomaly is genetic expression. There is evidence to support the relationship of several dental anomalies, including the delayed eruption, tooth size reduction, and abnormal shape of teeth, with the genetic factors [30]. Family studies have shown that both hypodontia and oligodontia are very likely to be inherited as an autosomal dominant trait with variable expression, and peg-shaped incisors are associated with agenesis of second premolars [31].

A previous study comparing the tooth size among different malocclusion types found no statistical difference between males and females, of Han Chinese decent (Beijing sample), in any malocclusion group [14]. However, the Class I group was either normal with no malocclusion or presented with bimaxillary protrusion. The anterior and overall Bolton ratios for this group of individuals from Beijing were found to be 81.52 ± 2.69 and 93.51 ± 2.46 while from the present study they were 77.04 ± 1.86 and 90.48 ± 1.79, respectively. This would indicate a difference in Bolton ratio between the Northern Chinese (Beijing sample) and Southern Chinese and Hong Kong individuals. The differences may be due to genetic variation between individuals North of the Yangtze River, i.e., individuals from Beijing, and those South of the river, i.e., Hong Kong, as genetically the populations are different [32]. These differences can be further confirmed by a study from Malaysia [33]. In that study, the anterior and overall Bolton ratios for Chinese were 76.55 ± 2.68 and 90.93 ± 1.87, respectively, which are very close to those in this study. This may be because the origin of the Chinese population in Malaysia is also from Southern China.

The result of this study supports the previous research finding that there is no sexual dimorphism of the anterior and overall Bolton's ratios in Class I occlusion [34].

Upon the consideration of tooth size discrepancy of contralateral incisors, canines, premolars, and molars of the same arch within the same gender, only the maxillary canines, lateral incisors, central incisor in males, and the mandibular canines and lateral incisors of females showed low levels of clinical significance. These are based on the calculation of Cohen's d (mean difference divided by standard deviation) between 0.2 and 0.5 [21].

Clinical application of Bolton's ratios enables orthodontists to plan the ideal aesthetic and functional outcomes of an orthodontic case without using a diagnostic setup. Orthodontists can also use it to assess the need for composite build up or tooth size reduction by interproximal stripping for those patients presenting with clinical significance in tooth size discrepancy [3, 35]. Because in some teeth the 95% confidence intervals for differences in mesiodistal between males and females were above 0.5 mm, even though the Bolton ratios were not different between males and females in this sample, individual tooth size and left-right asymmetry should also be taken into account as it was noted in both genders.

## 5. Conclusions

1. Females had statistically significant smaller teeth than males in southern Chinese except the upper left and lower left lateral incisor and lower left and right central incisors.
2. The anterior Bolton's ratios of males and females are 77.04 and 77.03, respectively, while the overall Bolton's ratios of male and female are 90.48 and 90.65, respectively. There were no significant gender differences in both ratios.
3. This study showed evidence that low level of clinical significant differences of the contralateral tooth size of maxillary canines, lateral incisors, and central incisors of southern Chinese male existed. On the other hand, low levels of clinical significant differences of contralateral tooth size of mandibular canines and lateral incisors were found in female.

## Figures and Tables

**Table 1:** Comparison of the mean tooth size, the mean anterior, and overall Bolton's ratio between male and female subjects by two-sample *t*-test.

Tooth	Male		Female		Mean Difference	SE of Mean Difference	95% Confidence Interval		*P* value
	Mean	SD	Mean	SD			Lower	Upper	
Maxillary									
T16	10.7	0.6	10.3	0.5	0.4	0.08	0.25	0.56	<0.001*∗*
T15	7.3	0.4	7.0	0.4	0.2	0.06	0.10	0.32	<0.001*∗*
T14	7.7	0.4	7.5	0.4	0.2	0.06	0.10	0.34	<0.001*∗*
T13	8.4	0.5	8.0	0.5	0.4	0.07	0.26	0.53	<0.001*∗*
T12	7.3	0.5	7.2	0.6	0.2	0.08	0.00	0.30	0.05
T11	8.7	0.5	8.5	0.5	0.2	0.07	0.08	0.36	0.003*∗*
T21	8.7	0.5	8.5	0.5	0.2	0.07	0.03	0.31	0.02*∗*
T22	7.3	0.5	7.1	0.5	0.1	0.07	0.00	0.29	0.054
T23	8.3	0.5	7.9	0.5	0.3	0.06	0.22	0.47	<0.001*∗*
T24	7.8	0.4	7.5	0.4	0.2	0.06	0.10	0.34	<0.001*∗*
T25	7.2	0.4	7.0	0.4	0.2	0.06	0.09	0.32	<0.001*∗*
T26	10.7	0.5	10.3	0.5	0.4	0.08	0.25	0.56	<0.001*∗*
Mandibular									
T36	11.4	0.5	11.0	0.6	0.4	0.08	0.25	0.55	<0.001*∗*
T35	7.5	0.5	7.3	0.5	0.2	0.07	0.08	0.36	0.002*∗*
T34	7.6	0.4	7.4	0.4	0.2	0.06	0.04	0.28	0.01*∗*
T33	7.2	0.4	6.9	0.4	0.3	0.06	0.21	0.44	<0.001*∗*
T32	6.1	0.4	6.0	0.4	0.1	0.05	-0.02	0.19	0.11
T31	5.5	0.4	5.4	0.4	0.1	0.05	-0.03	0.17	0.157
T41	5.5	0.4	5.4	0.4	0.1	0.05	-0.01	0.19	0.073
T42	6.1	0.4	6.0	0.4	0.1	0.05	0.02	0.22	0.022*∗*
T43	7.2	0.4	6.8	0.4	0.4	0.06	0.28	0.50	<0.001*∗*
T44	7.6	0.4	7.4	0.4	0.2	0.06	0.06	0.29	0.004*∗*
T45	7.5	0.5	7.3	0.5	0.2	0.07	0.06	0.33	0.004*∗*
T46	11.4	0.6	11.0	0.6	0.4	0.08	0.24	0.55	<0.001*∗*
Anterior ratio	77.04	1.86	77.03	1.86	0.01	0.26	-0.51	0.52	0.987
Overall ratio	90.48	1.79	90.65	1.60	0.17	0.24	-0.64	0.31	0.486

*∗P* < 0.05; SD = standard deviation; SE = standard error.

**Table 2:** Comparison of antimetric tooth size within the same gender by paired *t*-test.

Tooth pair	Mean	SD	95% Confidence Interval		Cohen's d Effect size	Clinical significance
	difference		Lower	Upper		
Male						
T16-T26	0.01	0.34	-0.06	0.07	0.01	< 0.2
T15-T25	0.03	0.22	-0.01	0.08	0.14	< 0.2
T14-T24	0.02	0.18	-0.05	0.02	0.08	< 0.2
T13-T23	0.10	0.26	0.05	0.15	0.39	> 0.2*∗*
T12-T22	0.05	0.21	0.01	0.09	0.24	> 0.2*∗*
T11-T21	0.06	0.20	0.02	0.10	0.29	> 0.2*∗*
T36-T46	0.00	0.26	-0.05	0.06	0.01	< 0.2
T35-T45	0.04	0.24	-0.01	0.08	0.16	< 0.2
T34-T44	0.01	0.21	-0.05	0.04	0.03	< 0.2
T33-T43	0.01	0.21	-0.06	0.03	0.07	< 0.2
T32-T42	0.02	0.17	-0.02	0.05	0.11	< 0.2
T31-T41	0.01	0.17	-0.03	0.04	0.04	< 0.2
Female						
T16-T26	0.00	0.23	-0.04	0.05	0.01	< 0.2
T15-T25	0.03	0.20	-0.01	0.07	0.14	< 0.2
T14-T24	0.02	0.22	-0.06	0.03	0.08	< 0.2
T13-T23	0.05	0.27	-0.01	0.10	0.18	< 0.2
T12-T22	0.05	0.25	-0.01	0.10	0.18	< 0.2
T11-T21	0.01	0.16	-0.02	0.04	0.07	< 0.2
T36-T46	0.00	0.24	-0.04	0.05	0.02	< 0.2
T35-T45	0.01	0.27	-0.04	0.06	0.03	< 0.2
T34-T44	0.01	0.23	-0.04	0.06	0.04	< 0.2
T33-T43	0.05	0.20	0.01	0.09	0.25	> 0.2*∗*
T32-T42	0.05	0.17	0.02	0.09	0.31	> 0.2*∗*
T31-T41	0.03	0.15	0.00	0.06	0.19	< 0.2

*∗*Cohen's d > 0.2 = clinical significance; SD = standard deviation.

## Data Availability

The data used to support the findings of this study are available from the corresponding author upon request.
